# Synergistic Induction of Chicken Antimicrobial Host Defense Peptide Gene Expression by Butyrate and Sugars

**DOI:** 10.3389/fmicb.2021.781649

**Published:** 2021-12-09

**Authors:** Qing Yang, Li-An Fong, Wentao Lyu, Lakshmi T. Sunkara, Kan Xiao, Guolong Zhang

**Affiliations:** ^1^Department of Animal and Food Sciences, Oklahoma State University, Stillwater, OK, United States; ^2^State Key Laboratory for Managing Biotic and Chemical Threats to the Quality and Safety of Agro-Products, Institute of Agro-Product Safety and Nutrition, Zhejiang Academy of Agricultural Sciences, Hangzhou, China; ^3^Veterinary Diagnostic Center, Clemson University, Clemson, SC, United States; ^4^Hubei Key Laboratory of Animal Nutrition and Feed Science, Hubei Collaborative Innovation Center for Animal Nutrition and Feed Safety, Wuhan Polytechnic University, Wuhan, China

**Keywords:** antimicrobial resistance, antibiotic alternatives, host defense peptides, antimicrobial peptides, sugar, monosaccharide, lactose, butyrate

## Abstract

Antimicrobial resistance is a major concern to public health demanding effective alternative strategies to disease control and prevention. Modulation of endogenous host defense peptide (HDP) synthesis has emerged as a promising antibiotic alternative approach. This study investigated a potential synergy between sugars and butyrate in inducing HDP gene expression in chickens. Our results revealed that sugars differentially regulated HDP expression in both gene- and sugar-specific manners in chicken HD11 macrophage cells. Among eight mono- and disaccharides tested, all were potent inducers of avian β-defensin 9 (*AvBD9*) gene (*p*<0.05), but only galactose, trehalose, and lactose obviously upregulated cathelicidin-B1 (*CATHB1*) gene expression. The expression of *AvBD14* gene, on the other hand, was minimally influenced by sugars. Moreover, all sugars exhibited a strong synergy with butyrate in enhancing *AvBD9* expression, while only galactose, trehalose, and lactose were synergistic with butyrate in *CATHB1* induction. No synergy in *AvBD14* induction was observed between sugars and butyrate. Although lactose augmented the expression of nearly all HDP genes, its synergy with butyrate was only seen with several, but not all, HDP genes. Mucin-2 gene was also synergistically induced by a combination of lactose and butyrate. Furthermore, lactose synergized with butyrate to induce *AvBD9* expression in chicken jejunal explants (*p*<0.05). Mechanistically, hyper-acetylation of histones was observed in response to both butyrate and lactose, relative to individual compounds. Mitogen-activated protein kinase, NF-κB, and cyclic adenosine monophosphate signaling pathways were also found to be involved in butyrate- and lactose-mediated synergy in *AvBD9* induction. Collectively, a combination of butyrate and a sugar with both HDP-inducing and barrier protective activities holds the promise to be developed as an alternative to antibiotics for disease control and prevention.

## Introduction

Routine use of antibiotics at subtherapeutic levels in feed for animal growth promotion and disease prophylaxis has been linked to the emergence of antibiotic-resistant bacteria in humans (Manyi-Loh et al., 2018; Mcewen and Collignon, 2018). It is a global trend to phase out in-feed antibiotics, necessitating the development of alternatives to antibiotics to maintain the productivity and health of food-producing animals. Host defense peptides (HDPs), also known as antimicrobial peptides, constitute an essential component of the innate immunity system (Robinson et al., 2015; Hancock et al., 2016; Ting et al., 2020). Dietary modulation of endogenous HDP synthesis has the potential to be developed as a novel antibiotic-free approach to disease control with a minimum risk of triggering antimicrobial resistance (Lyu et al., 2015; Robinson et al., 2018; Bergman et al., 2020; Rodriguez-Carlos et al., 2021).

In vertebrate animals, defensins and cathelicidins represent two major families of HDPs (Zhang and Sunkara, 2014; Rodriguez-Carlos et al., 2021). A total of 14 β-defensins known as AvBD1-14 and four cathelicidins (CATH1-3 and CATHB1) have been reported in chickens (Zhang and Sunkara, 2014). HDPs are produced in myeloid and/or epithelial cells lining the digestive, respiratory, and urogenital tracts (Zhang and Sunkara, 2014; Hancock et al., 2016). HDPs exert direct antimicrobial activities against Gram-negative and Gram-positive bacteria, fungi, and enveloped viruses with a minimum of risk triggering resistance. HDPs also play a range of immunomodulatory roles such as chemotaxis, activation of immune cells, and modulation of inflammation and autophagy (Hancock et al., 2016; Robinson et al., 2018). Additionally, HDPs contribute to maintaining epithelial homeostasis by inducing mucins and tight junction proteins (Robinson et al., 2015). Down-regulation of HDPs is employed by certain pathogens to evade host defense and establish infections, while stimulation of endogenous HDP synthesis has shown potential for antimicrobial therapy (Hancock et al., 2016; Robinson et al., 2018; Bergman et al., 2020; Rodriguez-Carlos et al., 2021). Several classes of compounds such as histone deacetylase inhibitors (HDACi), short-chain fatty acids, and vitamin D_3_ have been shown to promote HDP synthesis without triggering inflammation (Lyu et al., 2015; Bergman et al., 2020; Rodriguez-Carlos et al., 2021).

Butyrate is a major species of short-chain fatty acids produced by bacterial fermentation of undigested dietary fibers (Liu et al., 2018). Butyrate induces HDPs in humans, chickens, cattle, and pigs (Robinson et al., 2018; Rodriguez-Carlos et al., 2021) and confers protection against infections (Sunkara et al., 2011; Xiong et al., 2016). Butyrate induces HDPs by acting mainly as an HDACi to increase acetylation of core histones and relaxation of the target gene promoter (Kida et al., 2006; Robinson et al., 2018), and mitogen-activated protein kinase (MAPK) signaling pathways are also involved (Schauber et al., 2003). Sugars are represented by a group of dietary carbohydrates containing 1–2 monomeric sugar units and include monosaccharides, disaccharides, and sugar alcohols (Cummings and Stephen, 2007). Glucose has been found to enhance human β-defensin 1 (*DEFB1*) gene expression in keratinocytes (Cruz Diaz et al., 2015) and renal cells (Malik and Al-Kafaji, 2007), but decrease *DEFB3* and *DEFB4* in keratinocytes (Lan et al., 2011, 2012) and human cathelicidin antimicrobial peptide (*CAMP*) gene in monocyte-derived macrophages (Montoya-Rosales et al., 2016). However, lactose, a disaccharide derived from condensation of galactose and glucose, is capable of inducing the *CAMP* gene in human intestinal epithelial cells that involves MAPK, but not cyclic adenosine 3,5-monophosphate (cAMP) signaling (Cederlund et al., 2013). The induction of *CAMP* expression is also observed with several other mono- and disaccharides in the same study (Cederlund et al., 2013). Moreover, lactose is synergistic with butyrate in *CAMP* induction (Cederlund et al., 2013). The impact of lactose and its potential synergy with butyrate in HDP synthesis in other animal species and particularly non-mammalian species such as poultry remains unknown. Species-specific induction of HDPs in fact exists. For example, vitamin D_3_ is a potent inducer for human *CAMP* gene (Wang et al., 2004), but has a minimum ability to induce HDP expression in chickens (Zhang et al., 2016) and loses its activity completely in mice (Gombart et al., 2005).

To evaluate whether lactose can activate HDP gene expression and whether the synergy exists between lactose and butyrate in chickens, we studied the expression of three representative chicken HDP genes including *AvBD9*, *AvBD14*, and *CATHB1* in chicken HD11 macrophage cells in response to lactose and butyrate individually and in combination. We further extended our study to a panel of eight different mono- and disaccharides for their HDP-inducing ability and their synergy with butyrate in chicken cells. Additionally, the mechanisms by which butyrate and lactose induce *AvBD9* gene expression were examined.

## Materials and Methods

### Culture and Stimulation of Chicken HD11 Cells

Chicken HD11 macrophage cells (Sunkara et al., 2011, 2014) were maintained in complete RPMI 1640 medium (HyClone, Logan, UT, United States) containing 10% fetal bovine serum (Atlanta Biologicals, Flowery Branch, GA, United States), 100U/ml penicillin, and 100μg/ml streptomycin (Lonza, Walkersville, MD, United States). After overnight seeding at 2×10^6^ cells/well in 6-well cell culture plates, cells were stimulated with 0.1M or 0.2M of various sugars in the presence or absence of 2mM sodium butyrate (all from MilliporeSigma, St. Louis, MO, United States) for 3, 6, 12, 24, or 48h, followed by RNA isolation and gene expression analysis as described below. To study the role of different signaling pathways in HDP induction, 50μM PD98059, 20μM SP600125, 25μM SB203580, 0.1μM QNZ, or 1mm SQ22536 were incubated with cells 1h prior to treatment with 2mM butyrate and/or 0.2M lactose for 24h. To further probe the involvement of the Ras-Raf-MEK-ERK-RSK pathway, HD11 cells were pretreated with specific inhibitors including BAY43 (10, 20, and 40μM), U0126 (5, 10, 20, and 40μM), PD98059 (25, 50, 100, and 200μM), AG126 (25, 50, and 100μM), or SL0101 (10, 20, and 40μM) for 1h prior to stimulation with 2mM butyrate for 24h. All inhibitors were purchased from Santa Cruz Biotechnology (Dallas, TX, United States) or Cayman Chemical (Ann Arbor, Michigan, United States) and dissolved in dimethyl sulfoxide (DMSO).

### Preparation, Culture, and Stimulation of Chicken Jejunal Explants

The jejunal segments were harvested from 1- to 2-week-old broiler chickens, washed, cut into a series of 0.5-cm-long segments, and then cultured individually in 6-well plates as we previously described (Sunkara et al., 2014; Lyu et al., 2018). Each segment was stimulated with 0.1M lactose with or without 2mM butyrate and incubated in a Hypoxia Chamber (StemCell Technologies, Vancouver, BC, Canada) filled with 95% O_2_ and 5% CO_2_ at 37°C for 24h. Jejunal segments were then centrifuged and homogenized in RNAzol RT for RNA extraction.

### Quantitative Reverse Transcription-PCR

HD11 cells were lysed, and jejunal explants were homogenized in RNAzol RT (Molecular Research Center, Cincinnati, OH, United States) for extraction of total RNA. The first-strand cDNA was synthesized from 300ng of total RNA using Maxima^®^ First Strand cDNA Synthesis Kit (Thermo Fisher Scientific, Pittsburgh, PA, United States) in 4μl. Real-time PCR was then performed using QuantiTech^®^ SYBR Green I PCR kit (Qiagen, Valencia, CA, United States) and MyiQ Real-Time PCR Detection System (Bio-Rad, Hercules, CA, United States) in 10-μl reactions containing 1/10 of the first-strand cDNA and gene-specific primers for *AvBD1–10*, *AvBD14*, *CATHB1*, mucin-2 (*MUC2*), or claudin 1 (*CLDN1*). PCR cycling conditions were 95°C for 10min, followed by 40cycles of 94°C for 15s, 55°C for 20s, and 72°C for 30s. The specificity of PCR reactions was confirmed by the melting curve analysis. The gene expression levels were quantified using the comparative ΔΔCt method with the glyceraldehyde-3-phosphate dehydrogenase (*GAPDH*) gene as a reference for data normalization as described (Sunkara et al., 2011, 2014). Primers for chicken HDP genes and *GAPDH* used in the current study were as previously described (Sunkara et al., 2011). The primers for *MUC2* were TCTGGAGAGAGTTGTCCTGAC (forward) and TCCTTGCAGCAGGAACAACT (reverse), while TTCCAACCAGGCTTTATGATG (forward) and TGCAGAGTCAGGTCAAACAGA (reverse) were used for *CLDN1*.

### Western Blot Analysis

Chicken HD11 cells were stimulated with 0.2M lactose in the presence or absence of 2mM butyrate for 6, 12, or 24h, followed by wash with phosphate buffered saline and lysis in the radioimmunoprecipitation (RIPA) lysis buffer (Santa Cruz Biotechnology). Protein concentration was measured using the Bradford Assay (Bio-Rad). To determine the levels of histone H4 acetylation, 20μg proteins were separated in 12.5% SDS-PAGE gels and then transferred to polyvinylidene difluoride (PVDF) membranes. After overnight blocking in the blocking buffer containing 5% dry skim milk in TTBS (0.05% Tween 20, 20mm Tris-HCl, 150mm NaCl, pH 7.5) at 4°C, the membranes were incubated with a primary rabbit antibody against acetyl-histone H4 (Cell Signaling, Danvers, MA, United States) or a rabbit antibody against β-actin (MilliporeSigma) in the blocking buffer for 1h at room temperature. After three washes in TTBS, the membrane was incubated with an alkaline phosphatase-conjugated goat anti-rabbit IgG antibody (MilliporeSigma) for 45min at room temperature. Western blots were visualized with Western Blotting Luminol Reagent (Santa Cruz Biotechnology).

### Statistical Analysis

Statistical analysis and data visualization were implemented in GraphPad Prism (GraphPad Software, La Jolla, CA, United States). The results were expressed as means±standard error of the mean (SEM) from 2 to 3 independent experiments. Statistics was performed with one-way ANOVA and *post hoc* Tukey’s test. The results were considered statistically significant if *p*<0.05.

## Results

### Time- and Concentration-Dependent Induction of HDP Genes by Sugars

**Figure 1:**
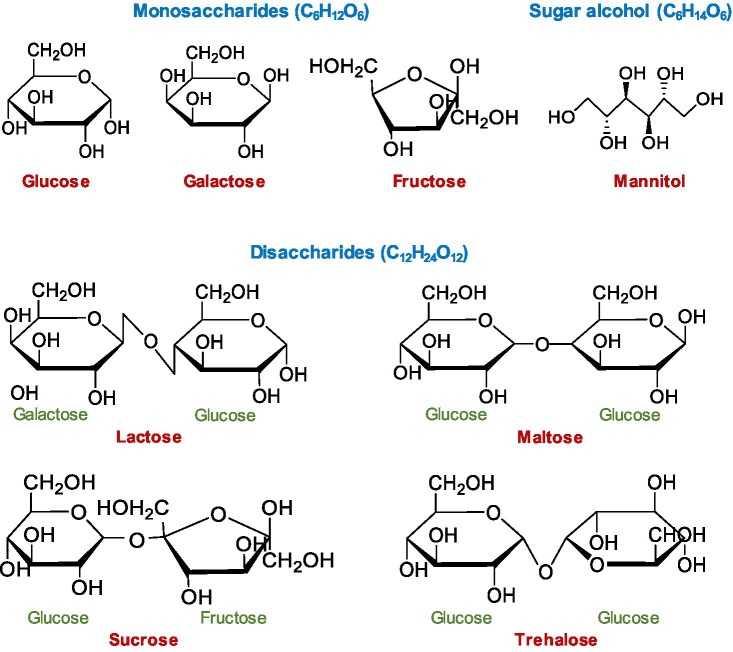
Chemical structure of mono- and disaccharide sugars and sugar alcohol tested in this study. Haworth projections of the chemical structures were drawn using ChemDraw JS (https://chemdrawdirect.perkinelmer.cloud/js/sample/index.html).

**Figure 2:**
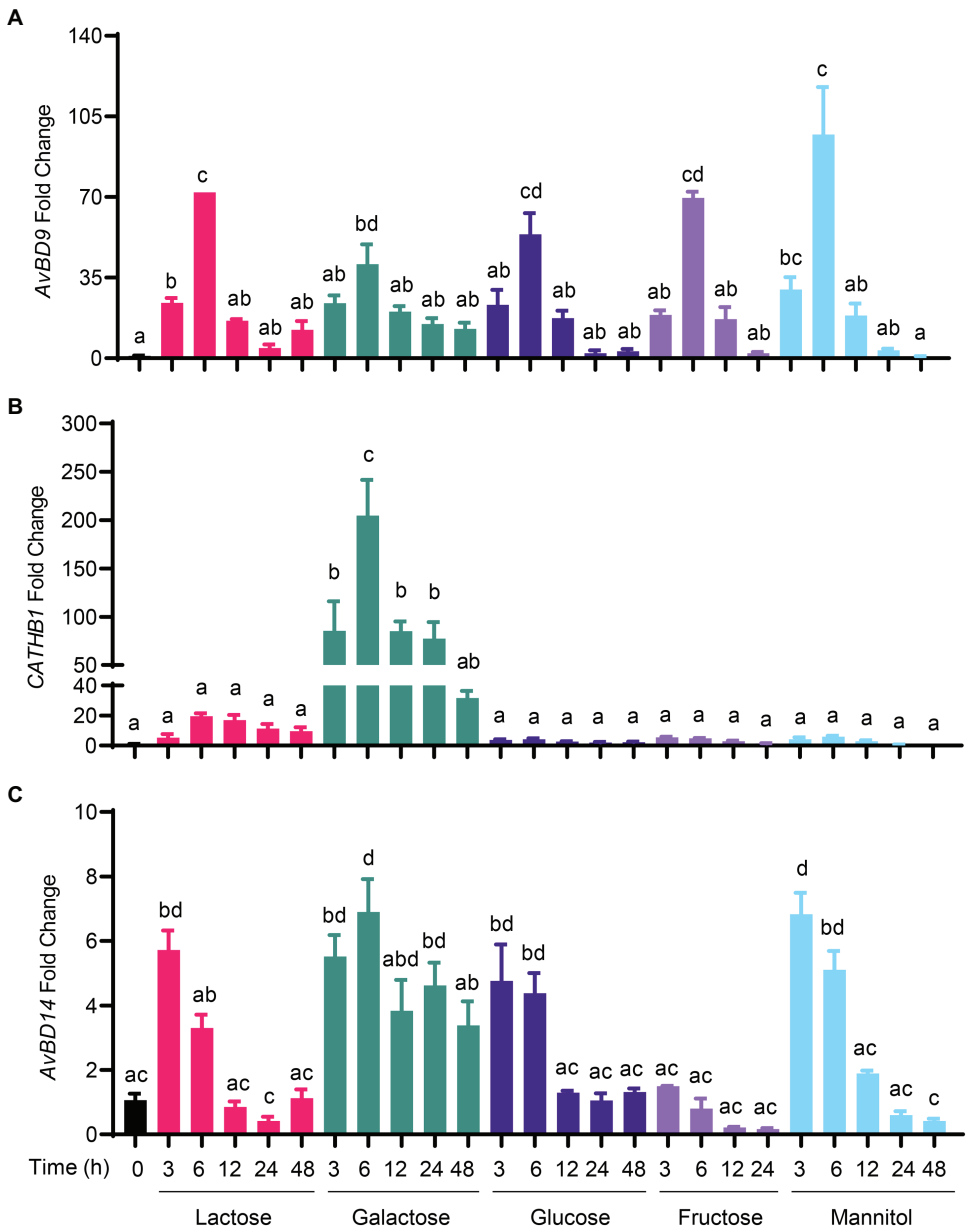
Time-dependent induction of host defense peptide (HDP) expression by sugars. Chicken HD11 macrophages were stimulated in duplicate with 0.2M of indicated sugars for 3, 6, 12, 24, or 48h. Cells were then subjected to RNA isolation and RT-qPCR analysis of gene expressions of avian β-defensin 9 (*AvBD9*) **(A)**, cathelicidin-B1 (*CATHB1*) **(B)**, and *AvBD14* **(C)**. Results are expressed as means±SEM of 2–3 independent experiments. The bars without common superscript letters denote statistical significance (*p*<0.05) as determined by one-way ANOVA and *post hoc* Tukey’s test.

To determine whether chicken HDP genes are induced by sugars, chicken HD11 macrophages were treated with three monosaccharides (glucose, galactose, and fructose), four disaccharides (lactose, maltose, sucrose, and trehalose), and a sugar alcohol (mannitol; Figure 1). All sugars at 0.2M clearly stimulated *AvBD9* gene expression in a time-dependent manner. *AvBD9* was readily induced as early as 3h, peaked at 6h, and then gradually declined to nearly basal levels at 24 and 48h (Figure 2A). All five sugars including mannitol showed a comparable efficacy in *AvBD9* induction, each with a peak response of an approximately 50- to 100-fold increase. Apparently, sugar-mediated HDP induction was gene-specific. Although the kinetics of the *CATHB1* mRNA expression was similar to that of *AvBD9* with a peak response at 6h, different sugars showed a dramatic variation in their potency to induce *CATHB1*. Galactose led to a greater than 200-fold maximum increase in *CATHB1* expression at 6h, whereas lactose gave only approximately 20-fold induction, with glucose, fructose, and mannitol showing a minimum two-fold to five-fold peak induction (Figure 2B). In the case of *AvBD14*, a peak induction occurred as early as 3h for lactose, glucose, and fructose, while galactose had a maximum induction at 6h (Figure 2C). However, only a maximum of five-fold to eight-fold change was observed with lactose, galactose, glucose, and mannitol, with essentially no induction seen with fructose (Figure 2C).

**Figure 3:**
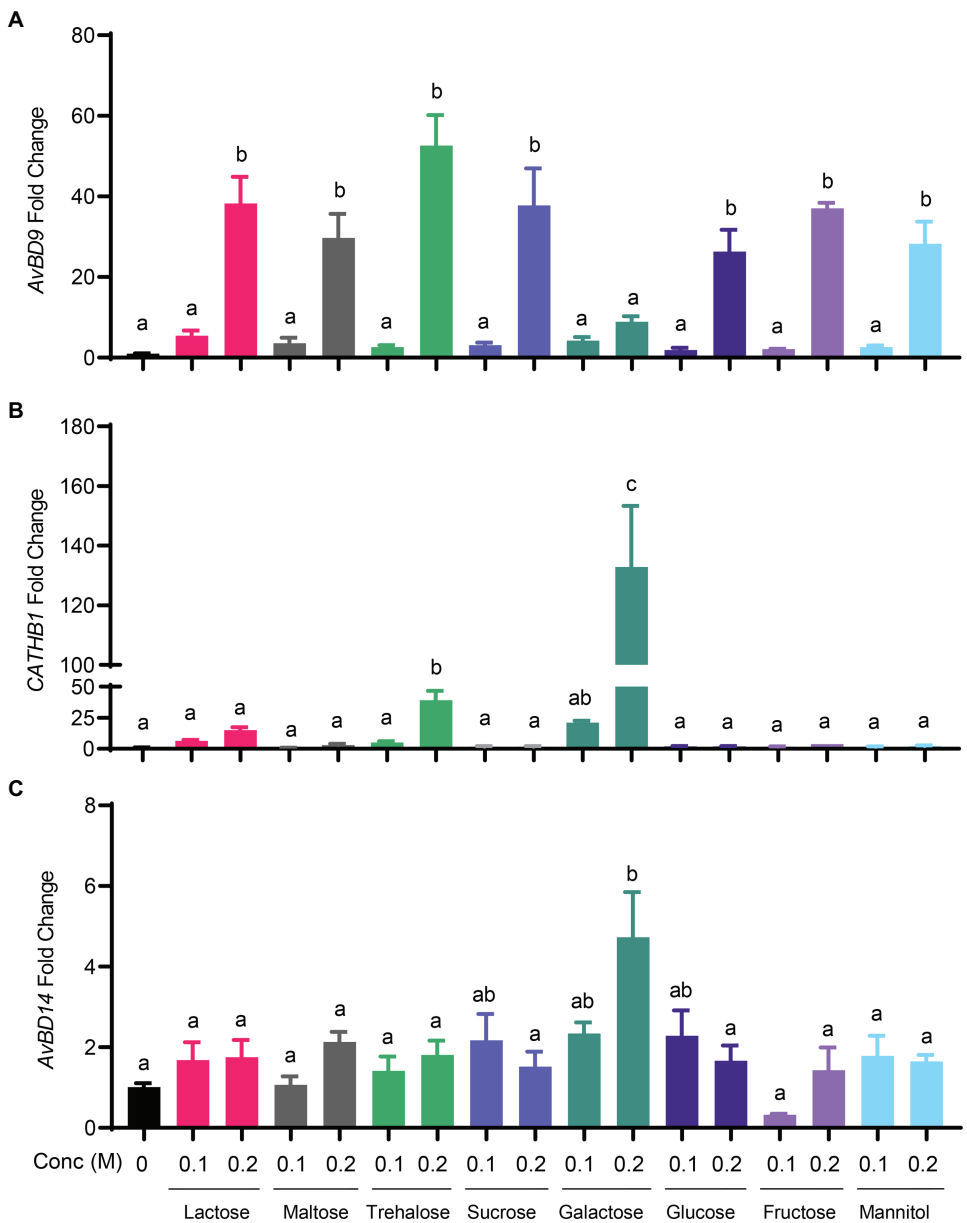
Dose-dependent induction of chicken HDP expression by sugars. Chicken HD11 macrophage cells were stimulated in duplicate with 0.1 or 0.2M of indicated sugars for 6h, followed by RNA isolation and RT-qPCR analysis of the gene expressions of *AvBD9* **(A)**, *CATHB1* **(B)**, and *AvBD14* **(C)**. Results are expressed as means±SEM of 2–3 independent experiments. The bars without common superscript letters denote statistical significance (*p*<0.05) as determined by one-way ANOVA and *post hoc* Tukey’s test.

Sugar-triggered *AvBD9* induction was also in a strong dose-dependent manner. All sugars at 0.1M showed a marginal effect on *AvBD9* induction at 6h, while 0.2M gave a substantial induction in HD11 cells (Figure 3A). As for *CATHB1*, only galactose, trehalose, and lactose triggered an obvious dose-dependent induction at 6h, with 0.2M galactose giving an approximately 150-fold increase, 0.2M trehalose giving an approximately 40-fold increase, and 0.2M lactose leading to a 20-fold induction (Figure 3B). Other sugars including maltose, glucose, sucrose, fructose, and mannitol had no or a negligible impact on *CATHB1* induction. *AvBD14* mRNA expression was minimally induced by most sugars, except for galactose causing an approximately five-fold increase at 0.2M (Figure 3C).

### Synergistic Induction of HDP and *MUC2* Gene Expression by Butyrate and Lactose

**Figure 4:**
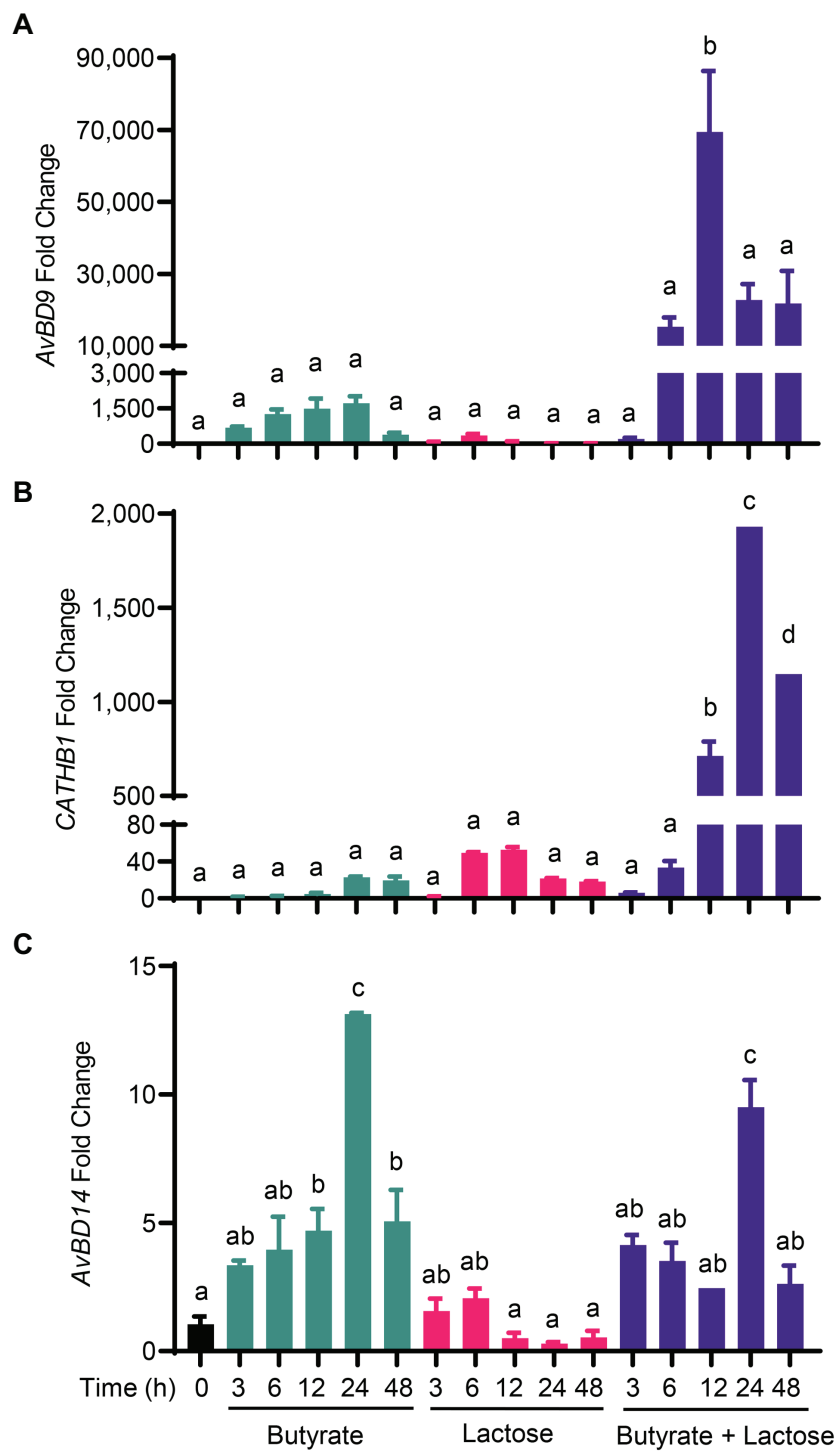
Synergistic induction of HDP expression by butyrate and lactose. Chicken HD11 macrophage cells were stimulated in duplicate with 2mM sodium butyrate and 0.2M lactose individually or in combination for 3, 6, 12, 24, or 48h, followed by RNA isolation and RT-qPCR analysis of the mRNA expression levels of *AvBD9* **(A)**, *CATHB1* **(B)**, and *AvBD14* **(C)**. Results are expressed as means±SEM of 2–3 independent experiments. The bars without common superscript letters denote statistical significance (*p*<0.05) as determined by one-way ANOVA and *post hoc* Tukey’s test.

To further explore a potential synergy between sugars and butyrate in HDP induction, we treated chicken HD11 cells with 2mM butyrate and 0.2M lactose individually or in combination for various lengths of time. As expected, butyrate triggered a peak response with an approximately 1,750-fold *AvBD9* induction at 24h, whereas lactose gave a maximum 350-fold *AvBD9* induction at 6h (Figure 4A). Desirably, a combination of butyrate and lactose resulted in synergistic enhancement of *AvBD9* expression at nearly all time points except for 3h. A peak response was seen at 12h with a nearly 70,000-fold increase, and the synergistic induction was sustained for at least 48h (Figure 4A). Butyrate and lactose also synergistically improved *CATHB1* transcription, peaking at 24h with a 2,000-fold induction (Figure 4B). Although butyrate and lactose enhanced *AvBD14* expression individually albeit at much lower magnitudes, no obvious synergy was observed when they were used together (Figure 4C), reinforcing the notion of differential regulation of HDP genes by lactose and butyrate.

**Figure 5:**
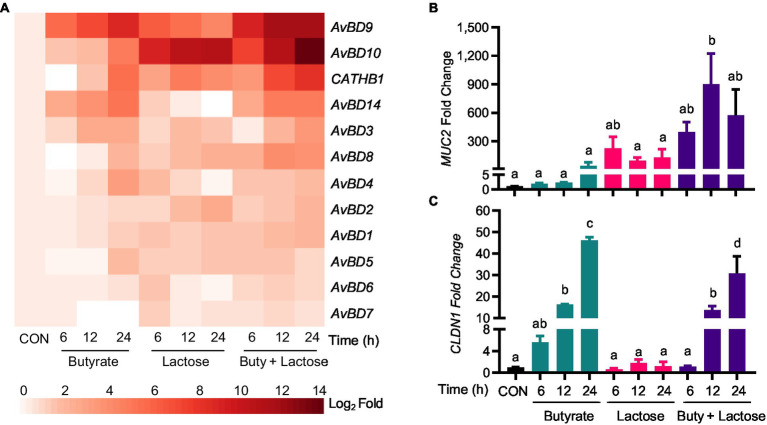
Synergistic induction of HDP and barrier function gene expression by butyrate and lactose. Chicken HD11 cells were stimulated in duplicate with 2mM sodium butyrate and 0.2M lactose individually or in combination for 6, 12, or 24h, followed by RNA isolation and RT-qPCR analysis of the mRNA expression levels of multiple HDP genes **(A)**, mucin 2 (*MUC2*) **(B)**, and claudin 1 (*CLDN1*) **(C)**. Results are expressed as means±SEM of two independent experiments. The bars without common superscript letters denote statistical significance (*p*<0.05) as determined by one-way ANOVA and *post hoc* Tukey’s test.

In addition, several other HDP genes such as *AvBD3*, *AvBD8*, and *AvBD10* were synergistically induced by a combination of butyrate and lactose (Figure 5A). For example, lactose alone strongly increased *AvBD10* expression and further synergized with butyrate to strengthen *AvBD10* transcription. A synergistic enhancement of *AvBD3* and *AvBD8* expressions was also observed at 12h or 24h. However, the remaining HDP genes were moderately affected by lactose and butyrate with no obvious synergy observed (Figure 5A). It is important to note that most HDP genes were induced by butyrate with a peak induction at 24h, but lactose induced HDP genes rather quickly peaking at 6h in many cases with a notable exception being *AvBD10*, which exhibited a time-depending increase with a maximum induction at 24h (Figure 5A). The strongest synergy occurred at 12 or 24h for most HDP genes.

Because of our interest in mucosal immunity and barrier integrity, two major genes involved in barrier function, namely *MUC2* and *CLDN1*, were also evaluated. Interestingly, lactose induced *MUC2* quickly showing a maximum induction in HD11 cells at 6h (Figure 5B). A clear synergy was also observed between butyrate and lactose in *MUC2* induction particularly at 12 and 24h (Figure 5B). On the other hand, *CLDN1* was prominently induced by butyrate, but not by lactose, and no obvious synergy was observed (Figure 5C).

**Figure 6:**
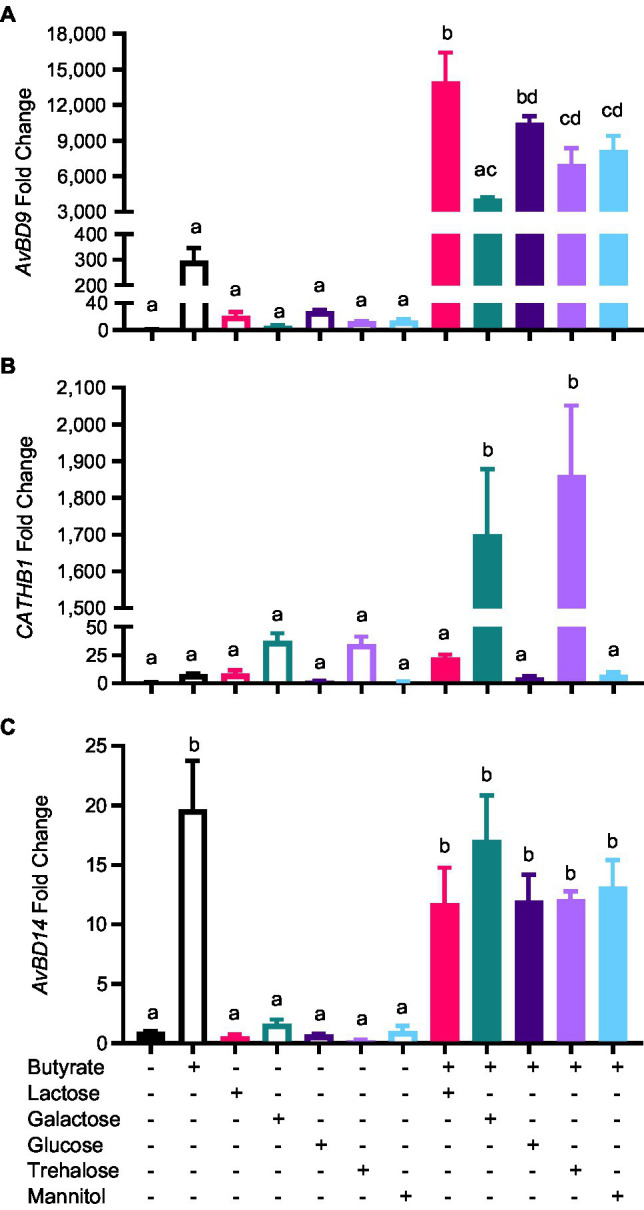
Synergistic induction of HDP expression by butyrate and sugars. Chicken HD11 cells were stimulated in duplicate with 2mM butyrate and 0.2M of indicated sugars separately or in combination for 12h, followed by RNA isolation and RT-qPCR analysis of the mRNA expression levels of *AvBD9* **(A)**, *CATHB1* **(B)**, and *AvBD14* **(C)**. Results are expressed as means±SEM of 2–3 independent experiments. The bars without common superscript letters denote statistical significance (*p*<0.05) as determined by one-way ANOVA and *post hoc* Tukey’s test.

To evaluate whether other mono- and disaccharides could also synergize with butyrate in improving HDP expression, HD11 cells were treated with butyrate and different sugars individually or in combination for 12h. Similarly, galactose, glucose, and trehalose showed a dramatic synergy in *AvBD9* induction (Figure 6A). Moreover, galactose and trehalose synergized with butyrate more strongly than lactose in *CATHB1* induction, whereas glucose and mannitol failed to induce *CATHB1* expression showing no synergy with butyrate (Figure 6B). In the case of *AvBD14*, none of the sugars investigated exhibited synergy with butyrate (Figure 6C).

**Figure 7:**
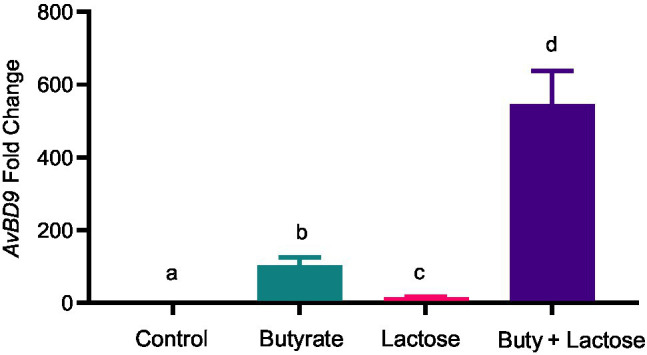
Synergistic induction of *AvBD9* gene expression in chicken jejunal explants by butyrate and lactose. Chicken jejunal explants were treated in duplicate with 2mM sodium butyrate and 0.1M lactose individually or in combination for 24h, followed by RNA isolation and RT-qPCR analysis of *AvBD9* expression. Results are shown as means±SEM of three independent experiments. The bars without common superscript letters denote statistical significance (*p*<0.05) as determined by one-way ANOVA and *post hoc* Tukey’s test.

**Figure 8:**
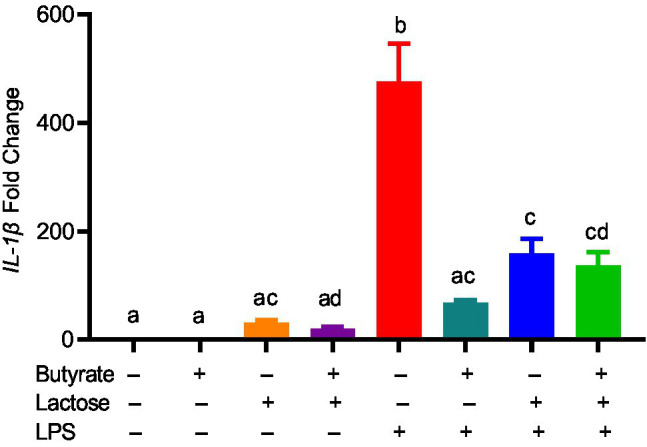
Suppression of lipopolysaccharide (LPS)-induced interleukin 1β (*IL-1β*) by butyrate and lactose. Chicken HD11 cells were treated in duplicate with 2mM butyrate, 0.1M lactose, or in combination for 1h, followed by stimulation with 10ng/ml LPS for another 3h and RT-qPCR measurement of *IL-1β* gene expression. The results presented means±SEM of two independent experiments. The bars without common superscript letters denote statistical significance (*p*<0.05) as determined by one-way ANOVA and *post hoc* Tukey’s test.

To confirm whether the HDP-inducing synergy between butyrate and sugars also occurs in other cell types, chicken jejunal explants were prepared and treated with butyrate and lactose separately or together. Butyrate (2mM) and lactose (0.1M) gave an approximately 120- and 20-fold induction of *AvBD9*, respectively, but more importantly, a marked synergy was observed, showing an approximately 550-fold increase in response to both compounds (Figure 7). To further examine how inflammatory response is affected by butyrate and lactose, HD11 cells were treated with butyrate or lactose individually or in combination in the presence or absence of bacterial lipopolysaccharide (LPS). As expected, butyrate was anti-inflammatory without altering interleukin-1β (*IL-1β*) expression; however, lactose caused a minimum induction of *IL-1β* expression (Figure 8). Importantly, lactose, butyrate, and the combination significantly reduced LPS-induced IL-1β expression, although no synergistic suppression was observed (Figure 8), suggesting an overall anti-inflammatory effect of the butyrate/lactose combination.

### Role of Histone Acetylation and MAPK, NF-κB, and cAMP Signaling in HDP Gene Expression Induced by Butyrate and Lactose

**Figure 9:**
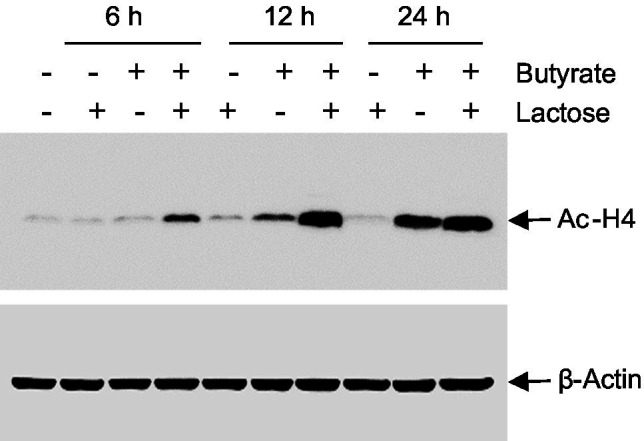
Acetylation of histone H4 in response to butyrate and lactose. Chicken HD11 cells were treated with 0.2M lactose with or without 2mM sodium butyrate for 6, 12, or 24h, followed by Western blot analysis for acetylation of histone H4. β-actin was also probed to show an equal amount of protein loading in each lane. The results are a representative of two independent experiments.

Butyrate induces HDP expression in chickens and humans mainly by acting as a HDACi (Kida et al., 2006; Robinson et al., 2018). To examine the impact of butyrate and lactose on histone acetylation, chicken HD11 cells were treated with butyrate and lactose individually or in combination for 6, 12, and 24h, followed by evaluation of the acetylation status of histone 4 (H4) using immunoblotting. As expected, butyrate triggered obvious H4 acetylation at 12h and the acetylation become more pronounced at 24h, while lactose had no impact on histone acetylation at any time point (Figure 9). A combination of both butyrate and lactose apparently accelerated and intensified histone acetylation, with H4 acetylation occurring evidently as early as 6h, peaking at 12h, and sustained at 24h.

**Figure 10:**
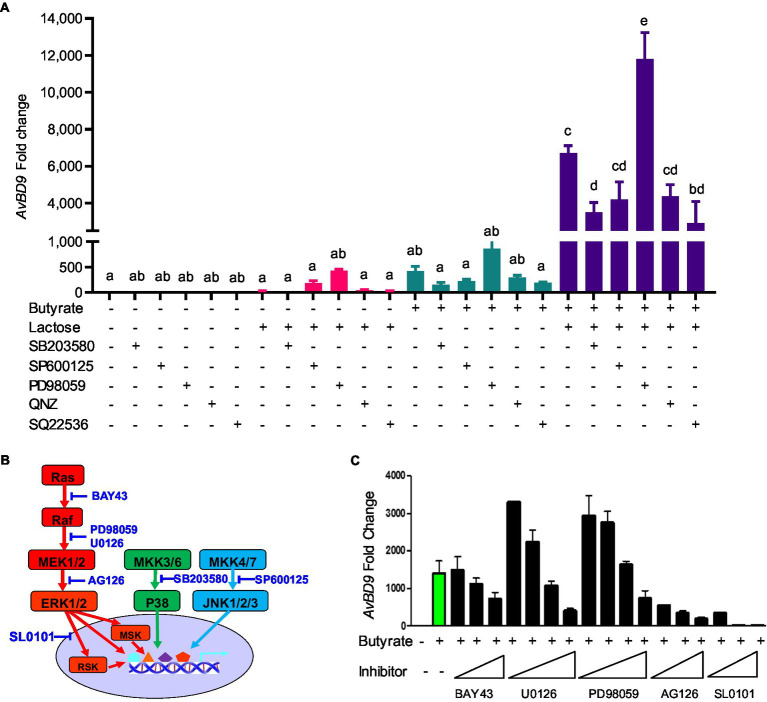
Involvement of mitogen-activated protein kinase (MAPK), NF-κB, and cAMP signaling pathways in *AvBD9* induction mediated by butyrate and lactose. **(A)** Chicken HD11 cells were pretreated for 1h with or without a specific inhibitor for p38 MAPK (SB203580), JNK (SP600125), MEK1/2 (PD98059), NF-κB (QNZ), or cAMP signaling (SQ22536), followed by stimulation with 2mM sodium butyrate and 0.2M lactose separately or in combination for another 24h. **(B)** A schematic drawing of three canonical MAPK pathways showing the steps where specific inhibitors act. **(C)** HD11 cells were pretreated for 1h with or without an indicated inhibitor for the Ras-Raf-MEK-ERK-RSK pathway, followed by stimulation with 2mM sodium butyrate for another 24h. RT-qPCR analysis of *AvBD9* expression was performed. Results are shown as means±SEM of 2–3 independent experiments. The bars without common superscript letters denote statistical significance (*p*<0.05) as determined by one-way ANOVA and *post hoc* Tukey’s test.

To examine the role of MAPK, NF-κB, and cAMP signaling pathways in butyrate- and lactose-mediated synergy in *AvBD9* induction, chicken HD11 cells were treated with butyrate and/or lactose for 24h in the presence or absence of specific inhibitors for each pathway. As expected, all inhibitors alone had a minimum influence on *AvBD9* gene expression, and inhibition of p38 MAPK (with SB203580), NF-κB (with QNZ), and cAMP pathways (with SQ22536) also had no influence on lactose-induced *AvBD9* expression (Figure 10A). On the other hand, inhibition of p38 MAPK, JNK (with SP600125), NF-κB, and cAMP pathways partially blocked butyrate-induced *AvBD9* expression, and the same four signaling pathways were similarly involved in *AvBD9* induction by a combination of butyrate and lactose (Figure 10A). Surprisingly, blocking the MAPK kinase 1/2 (MEK1/2) pathway by PD98059 significantly potentiated *AvBD9* gene expression induced by lactose, butyrate, or the combination (Figure 10A).

To further probe the involvement of the Ras-Raf-MEK1/2-ERK1/2-RSK pathway, inhibitors specific for kinases at different steps (Figure 10B) were employed. Consistently, inhibition of MEK1/2 with low concentrations of PD98059 and U0126 enhanced *AvBD9* gene expression, while increasing the concentrations of PD98059 and U0126 significantly suppressed *AvBD9* expression (Figure 10C). Inhibition of Raf with BAY43 also dose-dependently reduced *AvBD9* expression. The *AvBD9*-suppressing effect of blocking ERK1/2 and ribosomal S6 kinase (RSK) with AG126 and SL0101 was much more pronounced (Figure 10C). Collectively, these results suggested that, similar to the p38 MAPK and JNK pathways, the MEK-ERK pathway is also involved in butyrate-mediated HDP induction.

## Discussion

Sugars include mono- and disaccharides and constitute a group of dietary carbohydrates consisting of 1–2 simple sugar units, while oligosaccharides contain 3–9 sugar units, and polysaccharides contain >9 sugar units (Cummings and Stephen, 2007). All carbohydrates need to be broken down into monosaccharides to be utilized by animal hosts. Glucose and galactose are transported and taken up by the intestinal epithelial cells similarly through the involvement of sodium/glucose cotransporter 1 (SGLT1) and glucose transporter 2 (GLUT2), while GLUT2 and GLUT5 are involved in fructose transportation and absorption (Elferink et al., 2020). Disaccharides such as lactose, maltose, and trehalose lack the mechanism to be transported directed into intestinal epithelial cells and have to be broken down into two units of monosaccharides by different enzymes in the GI tract. Extended from an earlier study in humans (Cederlund et al., 2013), we have confirmed the conservation of the HDP-inducing activity of sugars in the chicken, a non-mammalian species, and further revealed a pronounced synergy between sugars and butyrate in augmenting chicken HDP expression. All eight most common sugars examined in this study including three monosaccharides, four disaccharides, and a sugar alcohol have the ability to induce the expression of certain HDP genes.

We have found that sugar-induced HDP expression is both gene- and sugar-specific. Among all chicken HDP genes, *AvBD9* and *AvBD10* are most readily inducible by lactose, while other genes are moderately or minimally induced. Among all sugars, *AvBD9* is uniformly induced, but other HDP genes exhibit a clear preference. For example, galactose is a potent inducer of *CATHB1* gene expression, while trehalose and lactose have a modest activity, and many other sugars are minimally active in *CATHB1* induction. On the other hand, *AvBD14* is minimally regulated by virtually all mono- and disaccharides tested. These results are consistent with an earlier study in humans, where trehalose is the most active in human *CAMP* gene induction in human HT-29 epithelial cells, with the potency gradually decreased in the order of maltose, lactose, glucose, and galactose (Cederlund et al., 2013). Gene-specific induction of HDPs was also observed with other small-molecule compounds such as butyrate (Sunkara et al., 2011, 2012; Zeng et al., 2013) and vitamin D_3_ (Zhang et al., 2016). For example, approximately a half number of chicken HDP genes are induced by butyrate, with *AvBD9* being the most inducible in chicken HD11 cells (Sunkara et al., 2012).

The synergy between butyrate and sugars also shows similar gene- and sugar-specific patterns of HDP regulation. All sugars synergize with butyrate in *AvBD9* induction with a similar potency; however, only galactose, trehalose, and lactose show a synergistic effect with butyrate in *CATHB1* induction, with galactose and trehalose giving much stronger synergy than lactose. It is noted that the kinetics of HDP induction by sugars or the sugar/butyrate combination is much different from most other HDP-inducing compounds. While it takes 24–48h for butyrate, fatty acids, HDACi, and vitamin D_3_ to achieve maximum HDP induction (Wang et al., 2004; Sunkara et al., 2011, 2012; Jiang et al., 2013; Zeng et al., 2013; Deng et al., 2018; Lyu et al., 2018), peak HDP expression occurs as early as 3–6h with a sugar.

All sugars tested in this study are capable of inducing *AvBD9* gene expression; however, the underlying mechanism remains largely unknown. An earlier human study revealed a partial involvement of the JNK and p38 MAPK pathways in lactose-induced human *CAMP* gene expression in HT-29 cells (Cederlund et al., 2013). However, neither MAPK pathway plays a critical role in lactose-mediated *AvBD9* induction. Such a discrepancy might be due to a difference in species, cell type, or HDP gene examined in the two studies. However, we have confirmed that all three canonical MAPK signaling pathways (JNK, p38 MAPK, and ERK1/2) are involved in the synergy in *AvBD9* gene induction by the lactose/butyrate combination. Although they are dispensable for lactose-induced *AvBD9* expression, NF-κB and cAMP signaling are partially responsible for the lactose/butyrate synergy, which is perhaps unsurprising, given the fact that sugars such as glucose are known to activate cAMP (Tengholm and Gylfe, 2017) and NF-κB (Kracht et al., 2020).

Butyrate is well known to induce HDP expression mainly by acting as an HDACi (Xiong et al., 2016; Rodriguez-Carlos et al., 2021). In this study, we have further revealed that although lactose has no direct impact on histone acetylation, histones are hyper-acetylated in response to a combination of lactose and butyrate. The exact mechanism is unknown, but it is tempting to speculate that lactose may act similarly as glucose, which has been extensively studied and shown to regulate gene expression through p300, also known as EP300 (Chen et al., 2010), a central transcriptional coactivator with histone acetyltransferase activity to enhance histone acetylation, chromatin relaxation, and gene transcription (Dancy and Cole, 2015). Glucose-mediated enhancement of p300 phosphorylation was recently shown to mediate through activation of 5′ adenosine monophosphate-activated protein kinase (AMPK) in a metabolic context-dependent manner (Gutierrez-Salmeron et al., 2020). AMPK is normally activated during glucose deprivation, but can also be activated in response to high glucose if glucose is blocked for glycogen synthesis inside a cell (Gutierrez-Salmeron et al., 2020).

It is worth noting that 25–50mM glucose was mostly used in the literature to activate AMPK or induce phosphorylation of p300. Much higher concentrations (0.1–0.2M) of glucose and other sugars have to be used in order to achieve optimal HDP induction in this study. The reason is unclear, but unlikely due to osmotic stress, because of the facts that: (1) up to 0.4M NaCl or KCl fails to induce human *CAMP* gene in HT-29 cells, whereas different sugars do so readily (Cederlund et al., 2013); (2) certain HDP genes such as *AvBD14* are barely induced by any sugar even at 0.2M (Figure 3); and (3) HDP genes such as *CATHB1* are only induced by few, but not all, sugars (Figures 2, 3). Mannitol, a non-metabolizable and membrane-impermeable sugar alcohol (Chen et al., 2020) induces chicken *AvBD9* gene and also synergizes with butyrate in *AvBD9* induction, similar to most other sugars, which is consistent with its ability to induce human *CAMP* gene (Cederlund et al., 2013). Additionally, chickens produce maltase and sucrase to break down sucrose and maltose, respectively, but appear to lack lactase to break down and take up lactose (Siddons, 1969; Siddons and Coates, 1972). Lactose is nevertheless still capable of inducing most HDP genes in chicken cells. These observations collectively suggest a presence of transmembrane receptors to mediate sugar-induced HDP expression. In fact, sugars and sweeteners can bind to ubiquitously expressed sweet taste receptors T1R2 and T1R3 to activate multiple complex signaling pathways in mammals (Lee and Owyang, 2017; Von Molitor et al., 2021) and also in avian species (Niknafs and Roura, 2018; Roura and Foster, 2018). It will be important to examine the involvement of taste receptors or other receptors in sugar-mediated HDP induction.

In addition to upregulating HDP expression, lactose and likely other sugars also strongly augment the gene expression of *MUC2*, which is in turn translated to the predominant mucin protein in the intestinal epithelium (Vancamelbeke and Vermeire, 2017; Liu et al., 2020). Butyrate is also well-known to induce *MUC2* (Bach Knudsen et al., 2018). The synergy between butyrate and lactose in inducing *MUC2* may potentiate their protection of the epithelial barrier. With an additional ability to suppress LPS-induced inflammatory response (Figure 8), butyrate and lactose have the potential to enhance gut health and alleviate infections. In fact, butyrate and 2.5% lactose have been separately used in chickens with a beneficial effect on alleviating infections such as necrotic enteritis (Mcreynolds et al., 2007; Liu et al., 2019). Although the mechanism of action was not studied earlier, augmenting HDP gene expression and barrier function while suppressing inflammation may be at least partially responsible for enhanced disease resistance in chickens fed butyrate or lactose. Although a combination of butyrate and lactose is yet to be tested, we recently found chickens supplemented with a mixture of 0.1% encapsulated sodium butyrate, 1% lactose, and 5–10ppm forskolin-containing plant extract to be protected from both necrotic enteritis and coccidiosis (Yang et al., 2021). Butyrate/forskolin-mediated protection of chickens from necrotic enteritis is also mediated through enhancing HDP expression and barrier function (Robinson et al., 2021).

This study is focused on mono- and disaccharides. The role of dietary oligosaccharides and polysaccharides in regulating HDP gene expression is largely unknown, except that two human milk oligosaccharides were recently found to increase human β-defensin 2 protein synthesis, but not other HDPs or inflammatory cytokines (Gursoy et al., 2021). It will be interesting to explore possible HDP-inducing and other immunomodulatory roles of commonly used dietary oligosaccharides and polysaccharides such as fructo-oligosaccharides, galacto-oligosaccharides, xylo-oligosaccharides, mannan-oligosaccharides, and inulin, all of which are being actively explored as prebiotics to manage the gut microbiome, gut health, and diseases (Zhu et al., 2019).

## Conclusion

Mono- and disaccharide sugars are capable of inducing the expressions of HDP genes in chicken HD11 macrophages and jejunal explants in gene- and sugar-specific manners. Moreover, these sugars synergize with butyrate to further enhance chicken HDP expression. Additionally, lactose is synergistic with butyrate in upregulating *MUC2* expression. Promoting histone hyper-acetylation is at least partially responsible for lactose- and butyrate-mediated synergy in *AvBD9* induction. MAPK, NF-κB, and cAMP signaling pathways are all involved in *AvBD9* expression induced by lactose and butyrate. Our results suggest a prospect for the development of a combination of sugars and butyrate as an antibiotic-alternative approach to infectious disease control and prevention.

## Data Availability Statement

The original contributions presented in the study are included in the article/supplementary material, further inquiries can be directed to the corresponding author.

## Author Contributions

QY, L-AF, WL, LTS, and KX conducted the experiments. QY, L-AF, and GZ performed data analysis. QY and L-AF drafted the manuscript. GZ conceived the study and revised the manuscript. All authors contributed to the article and approved the submitted version.

## Funding

This research was funded by the USDA National Institute of Food and Agriculture (grant nos. 2018-68003-27462 and 2020-67016-31619), Oklahoma Center for the Advancement of Science and Technology (grant no. AR19-027), the Ralph F. and Leila W. Boulware Endowment Fund, and Oklahoma Agricultural Experiment Station Project H-3112.

## Conflict of Interest

The authors declare that the research was conducted in the absence of any commercial or financial relationships that could be construed as a potential conflict of interest.

## Publisher’s Note

All claims expressed in this article are solely those of the authors and do not necessarily represent those of their affiliated organizations, or those of the publisher, the editors and the reviewers. Any product that may be evaluated in this article, or claim that may be made by its manufacturer, is not guaranteed or endorsed by the publisher.
